# Noninvasive Ventilation in Preterm Infants: Factors Influencing Weaning Decisions and the Role of the Silverman-Andersen Score

**DOI:** 10.3390/children9091292

**Published:** 2022-08-26

**Authors:** Claudia Nussbaum, Maximilian Lengauer, Alexandra F. Puchwein-Schwepcke, Veronique B. N. Weiss, Benedikt Spielberger, Orsolya Genzel-Boroviczény

**Affiliations:** 1 Division of Neonatology, Department of Pediatrics, Dr. von Hauner Children’s Hospital, University Hospital, LMU Munich, 80337 Munich, Germany; 2 Department of Pediatric Neurology and Developmental Medicin, University of Basel Children’s Hospital, 4056 Basel, Switzerland

**Keywords:** preterm, neonate, noninvasive ventilation, CPAP, weaning, Silverman Score

## Abstract

The factors influencing weaning of preterm infants from noninvasive ventilation (NIV) are poorly defined and the weaning decisions are often driven by subjective judgement rather than objective measures. To standardize quantification of respiratory effort, the Silverman-Andersen Score (SAS) was included in our nursing routine. We investigated the factors that steer the weaning process and whether the inclusion of the SAS would lead to more stringent weaning. Following SAS implementation, we prospectively evaluated 33 neonates born ≤ 32 + 0 weeks gestational age. Age-, weight- and sex-matched infants born before routine SAS evaluation served as historic control. In 173 of 575 patient days, NIV was not weaned despite little respiratory distress (SAS ≤ 2), mainly due to bradycardias (60% of days without weaning), occurring alone (40%) or in combination with other factors such as apnea/desaturations. In addition, “soft factors” that are harder to grasp impact on weaning decisions, whereas the SAS overall played a minor role. Consequently, ventilation times did not differ between the groups. In conclusion, NIV weaning is influenced by various factors that override the absence of respiratory distress limiting the predictive value of the SAS. An awareness of the factors that influence weaning decisions is important as prolonged use of NIV has been associated with adverse outcome. Guidelines are necessary to standardize NIV weaning practice.

## 1. Introduction

The majority of preterm neonates born < 32 weeks of gestation requires respiratory support [1]. Large randomized controlled trials have proven the benefit of early nasal continuous positive airway pressure (CPAP) over routine intubation with respect to the combined outcome of bronchopulmonary dysplasia and death [2,3]. Accordingly, current guidelines on the management of respiratory distress syndrome (RDS) recommend CPAP or other modes of non-invasive ventilation for primary respiratory support [4,5]. Noteworthy, the increased use of NIV observed over the last decades has led to a longer overall duration of positive pressure respiratory support for days to even weeks [6,7]. Despite the advantages of NIV, there are certain risks, including an increased rate of air leak syndromes, gastric distension, nasal trauma and secondary intubation due to respiratory exhaustion [3,8]. Moreover, prolonged use of NIV might have adverse effects on long-term pulmonary function [7]. The comparison of extreme preterm infants born at three different periods (2005, 1997 and 1991–1992) demonstrated a significantly longer use of CPAP and oxygen dependence in the 2005 cohort combined with higher rates of airway obstruction in lung function testing at the age of eight compared to the prior eras [7]. Therefore, NIV should be used no longer than necessary. With respect to NIV practice, considerable heterogeneity exists not only between different neonatal intensive care units but also between different health care providers [9]. The decision of if, when, and how to reduce NIV is often based on the subjective judgement of nurses and clinicians as standardized weaning guidelines are lacking.

The Silverman-Andersen Score (SAS) is an easy and inexpensive method to judge the degree of respiratory distress and the need for respiratory support in neonates [10,11,12]. The SAS has been shown to correlate with pCO2 levels and predicted the necessity to increase ventilatory assist [13]. We introduced the SAS to our nursing routine for all infants with NIV to standardize quantification of respiratory distress symptoms with the aim of providing a more objective estimate of the actual work of breathing and to serve as decision aid for the healthcare team during NIV weaning.

In the present study, we prospectively investigated which factors influence weaning decisions of health care professionals on the neonatal intensive care unit. We hypothesized that including the SAS into the weaning process would lead to more stringent weaning and thereby could possibly reduce NIV duration. Furthermore, we tested the interrater reliability of the SAS.

## 2. Materials and Methods

### 2.1. Patient Recruitment and Data Collection

Neonates ≤ 32 + 0 weeks gestational age (WGA) at birth were prospectively included in the study over a period of 16 months at the neonatal intensive care unit (NICU), Perinatal Center Innenstadt, Ludwig-Maximilians University, Munich, Germany after obtaining written informed consent. The study was approved by the local ethics committee. To account for the higher respiratory morbidity in more premature infants, especially during the immediate postnatal phase, data collection was not started until infants reached a corrected gestational age of ≥30 + 0 weeks in those infants born < 30 + 0 WGA. To be eligible, infants had to be on NIV for at least 48 h irrespective of the prior mode of ventilation. Exclusion criteria were asphyxia, congenital malformations, suspected or proven syndromes, bodyweight < 1000 g at 30 + 0 weeks, and retinopathy of prematurity (ROP) ≥ Grade 3. A historic (retrospective) collective of patients in our NICU before introducing the SAS served as a control group. To match controls for age, birthweight, and gender, patient records were screened from 2017 on backward before SAS implementation and the first matching record was included.

### 2.2. Noninvasive Ventilation

As we included the SAS in our existing clinical routine, NIV mode, interfaces, and respirators were not defined by the study but chosen by the attending neonatologist and applied according to our internal NICU standards (Supplemental Methods). There has been no relevant change in the NIV modes, interfaces, and respirators used between the prospective and the retrospective study period.

Briefly, the following NIV modes are applied on our ward: synchronized noninvasive positive pressure ventilation (sNIPPV), neutrally adjusted ventilatory assist (NAVA), continuous airway pressure (CPAP) and heated humidified high flow nasal cannula (HHHFNC). For synchronization of NIV with the child’s spontaneous breaths, an abdominal respiratory sensor was used (Fa. Fritz Stephan GmbH, Gackenbach, Germany) or a specialized oesophageal probe for NAVA (Getinge Deutschland GmbH, Rastatt, Germany). For sNIPPV, CPAP, and NAVA, the positive end-expiratory pressure (PEEP) level was directly set at the respirator, while for HHHFNC, the end-expiratory pressure was estimated according to the following formula [14]:

pressure (cmH_2_O) = 0.7 × 1.1 F (F = flow per kg in L min^−1^ kg^−1^).

Supplemental oxygen was titrated by the nurses according to a SpO_2_ target range of 88–95%.

### 2.3. Silverman-Andersen Score

The Silverman-Andersen Score has been implemented for routine evaluation of infants with NIV on our NICU, starting in mid-2017. For this purpose, nurses and physicians were instructed on the use of the SAS, and the scoring scheme was included in the charts available at every bed place. The SAS includes five clinical categories, i.e., sternal and intercostal retractions, nasal flaring, expiratory grunting, and upper chest movements, with 0–2 points for each category [13]. Zero points indicate normal respiration and ten points severe respiratory distress. The nurse in charge of the infant assigned and documented the SAS at least once per shift (=3 x/d) at the beginning of care rounds and irrespective of study participation. With respect to the SAS, the following categories were defined as an indicator of the child’s need of respiratory support. SAS = 0–2 points: no/mild respiratory distress; SAS = 3 points: moderate respiratory distress; SAS ≥ 4 points: significant respiratory distress.

### 2.4. Weaning Process

In our NICU, preterm neonates < 32 + 0 WGA are mostly started on NIPPV or NIV-NAVA and then weaned via CPAP and HHHFNC until NIV can be discontinued or switched to a low flow nasal cannula. Accordingly, the weaning process as analyzed in the present study comprises the whole period from initiation of NIV until its termination. No specific weaning protocol existed for the historical controls, and the weaning decision was at the attending physician’s discretion after team discussion. For the prospective cohort, the healthcare team decided during morning rounds whether the NIV could be reduced or not pursuing the following approach with respect to the SAS: (1) 0–2 points: reduce NIV, 3 points: maintain NIV, ≥4 points: increase NIV. Still, the team was allowed to deviate from this recommendation if they deemed other factors (e.g., increased rate of apneas, higher oxygen demand) to be more relevant. Our study did not include specific instructions on how NIV should be weaned.

### 2.5. Outcome Parameters

For every patient, NIV mode and parameters were noted daily as well as the reasons for a decision against weaning. The following definitions were used: bradycardia = heart rate < 80 bpm, hypoxemia = oxygen saturation < 80%, apnea = pause of spontaneous respiratory efforts for 20 s duration or 10 s with concomitant bradycardia and/or hypoxemia, tachypnea = respiratory rate > 80/min, increased FIO_2_ = demand of oxygen supply > 20% from baseline; discomfort = N-Pass score > +3. Clinical data (including anthropometric measurements at birth and at discharge from the clinic, the reason for preterm birth, birth mode, APGAR values, vital parameters, medications and occurrence of complications) as well as laboratory data (blood gases, blood counts and infectious parameters) were taken from the records.

We evaluated total NIV duration, NIV duration beyond 30 + 0 weeks corrected gestational age as well as the duration of different NIV modes and the gestational age at the end of NIV. Secondary outcome parameters were the length of supplemental oxygen need and hospital stay.

### 2.6. Interrater Reliability Testing

To compare the SAS between different raters, 45 video sequences (30–60 s) of 10 study patients were recorded by the nurses during care rounds in parallel to SAS scoring and assessed by a research assistant (M.L.), and a neonatologist (C.N.) blinded to the medical condition of the patient. The videos were stored under the patient’s pseudonym and the date of recording to be able to match scores of the two independent raters with the nurse’s score.

### 2.7. Statistics

GraphPad Prism 8.2 (GraphPad Software Inc., San Diego, CA, USA) was used for statistical analyses. After normality testing by the D’Agostino Pearson test, a *t*-test or a Mann-Whitney test were used to compare numerical data, as appropriate. A comparison of categorical data was made by Fisher’s exact test (2 variables) or by the Chi-Square test (3 variables). Interrater reliability was assessed by Fleiss’ kappa and by Bland-Altman Diagram. For numerical clinical data with normally distribution the mean ± standard deviation is reported. In the case of non-normally distributed data, the median and range is reported.

## 3. Results

### 3.1. Patients

**Figure 1:**
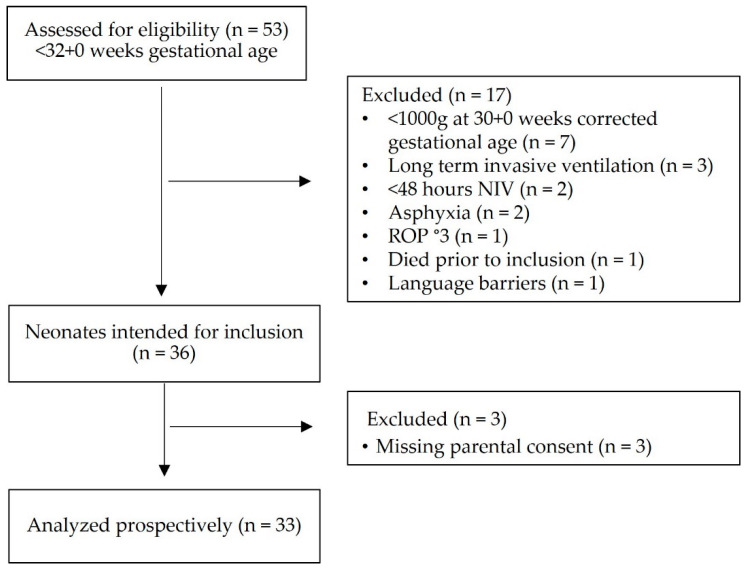
Consort flow diagram of prospective patient recruitment.

During the study period, 53 neonates ≤ 32 + 0 gestational weeks were admitted to our NICU. Of these, 17 were excluded (Figure 1), and of the remaining 36 neonates, we obtained parental consent in 33 and prospectively observed them over a total of 575 patient days (SAS group). Controls were identified retrospectively from 2017 backward until 2015 (control). Baseline characteristics and use of different NIV modes were comparable between both groups (Table 1). The two groups did not show significant differences in important determinants of respiratory outcome including antenatal steroids, use of surfactant and caffeine and primary intubation rate.

### 3.2. Analysis of the Weaning Process

**Figure 2:**
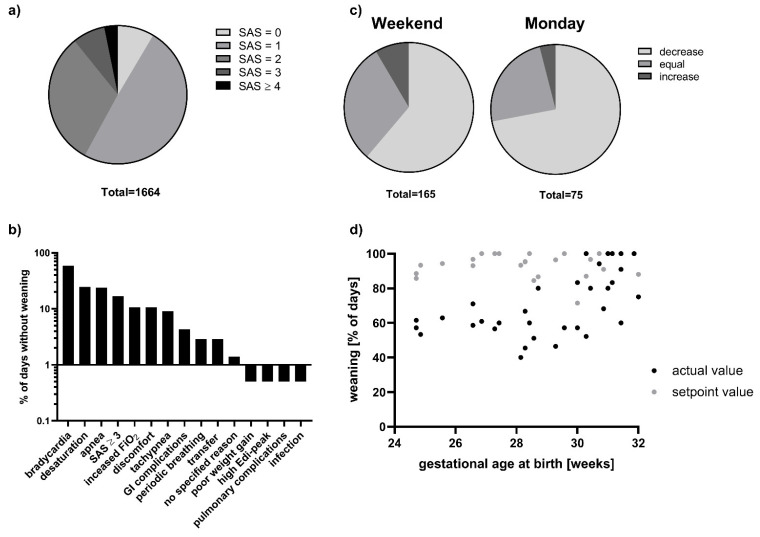
(**a**) SAS distribution in the prospective cohort. (**b**) Reasons for a decision against weaning. GI = gastrointestinal, Edi = electrical activity of the diaphragm. (**c**) Weaning on days of the weekend versus Mondays. (**d**) Actual and setpoint weaning per individual child.

NIV was weaned in 367 (64%) of 575 patient days observed. In the remaining 208 days, NIV parameters were kept the same (28%) or increased (8%). A total of 1664 SAS were available, with 89% being in the range of 0–2 points; only in 3% of all SAS, a score ≥ 4 points was assigned (Figure 2a). Looking at the days without weaning, a SAS > 2 was documented only in 17%. Thus, the healthcare team decided against weaning in 173 patient days (30% of total days) despite no or minor symptoms of respiratory distress. As shown in Figure 2b, we identified various factors that influenced weaning decisions. The most common reason for maintaining or enhancing NIV parameters was an increased incidence of bradycardias found in 59% of all days without weaning. In 40% of the time, bradycardias occurred alone; in the remaining 60%, there was a combination with other factors, mostly apnea (24%) and desaturations (25%). Other reasons for not weaning included tachypnea, increased oxygen demand, and discomfort of the child (Figure 2b). Interestingly, weaning differed between weekends and workdays. While infants were weaned on 72% of Mondays, NIV was only reduced in 61% of Saturdays/Sundays (Figure 2c). Furthermore, we evaluated for every individual child the percentage of days with weaning (actual value) compared to the percentage of days with SAS ≤ 2 (setpoint value). As shown in Figure 2d, extreme premature neonates were less likely to be weaned despite low SAS and comparable corrected gestational age at the beginning of the evaluation process than more mature neonates. With respect to the mode of weaning, we mostly observed pressure weaning (i.e., a reduction in the supporting peak pressure and/or the PEEP level), however towards the end of the weaning process on low level CPAP or HHHFNC, intermittent breaks from the respiratory support were introduced in some children in both cohorts.

Comparison of the SAS between three different raters (nurse, student, neonatologist) yielded only slight agreement with a Fleiss’ Kappa of 0.18 (*p* = 0.002). The poorest agreement was found between research assistant and neonatologist (Fleiss’ kappa 0.08). However, there was no systematic bias towards higher or lower scores in one particular rater (Supplemental Figure S1).

### 3.3. Effect of Routine SAS Assessment on NIV Outcome Parameters

NIV duration did not differ significantly between study groups, nor did any other outcome measure (Table 2). In the SAS group, median NIV duration beyond 30 + 0 gestational weeks was three days shorter than in the control group (17 vs. 20 days). Looking at different NIV modes, it became evident that this difference was only attributable to the time on HHHFNC, particularly with a PEEP < 3 cmH_2_O. The mean gestational age at NIV termination was 33 + 1 (wks + d) in the SAS group vs. 33 + 3 in the controls. Caffeine treatment was usually continued until NIV was terminated and we did not observe significant differences in maximum (maintenance) dose (7.9 ± 3.2 mg/kg vs. 8.6 ± 3.4 mg/kg) or treatment duration (33 ± 22 d vs. 35 ± 26 d) between patients and controls.

The incidence of complications was comparable in both groups (Table 3). Neither the higher occurrence of a persistent ductus arteriosus (PDA) in the SAS group nor the slightly more common grade 3 intraventricular hemorrhages (IVH) in the control group reached statistical significance.

## 4. Discussion

Noninvasive ventilation is used worldwide to support preterm infants with respiratory distress. While a large consensus exists about the clinical criteria that trigger initiation of NIV, the criteria indicating that an infant can be weaned are not well defined. Therefore, the weaning process is often based on individual judgement and the impression that the “baby is ready” rather than objective measures [15]. In an effort to standardize the evaluation of respiratory distress symptoms in neonates with NIV, we included the SAS in our clinical routine to help assess the infant’s actual work of breathing and ongoing need of respiratory support. We hypothesized that this might lead to more consistency in the weaning process and shorten times on ventilatory support. However, this was not confirmed by our data. We only observed a small difference of 2–3 days in the median duration of total NIV and ≥ 30 + 0 gestational weeks compared to a matched control group cared for on our NICU before the introduction of the SAS. In line with previous studies, the average age at the end of NIV was around 33 weeks corrected gestational age [16,17,18].

The results of our study illustrate that weaning decisions are influenced by many factors, some of which are easy to retrace, e.g., increased oxygen need, while others are more difficult to grasp (so-called “soft factors”). The most common reason to maintain or enhance NIV was an increased incidence of bradycardias occurring alone or in combination with apneas and desaturations. As CPAP helps to stabilize the upper airway and reduces the rate of apneas [19], it seems reasonable that the physicians are reluctant to reduce NIV in this situation. However, while intermittent hypoxia has been shown to negatively affect the neurological outcome and therefore should be avoided [20], the effect of isolated self-limited bradycardias without desaturation on patient outcome is questionable. Furthermore, isolated bradycardias are not necessarily associated with apnea but can also be an expression of vagal stimulation, e.g., due to gastroesophageal reflux or a sign of infection. As suggested by Doyle and colleagues, we might be “overusing” CPAP due to continuous monitoring of infants and the impulse to react to monitor events even if their significance is unclear [7]. This problem might be approached by using a score that grades the severity of apneas and associated bradycardias [21].

With respect to the SAS, elevated scores (≥3) were 100% predictive for a decision against weaning, however, they accounted only for 17% of days without weaning. Interestingly, our results do not support the reverse conclusion, i.e., that a low SAS will lead to weaning, as NIV was not reduced in 1/3 of patient days despite a SAS ≤ 2. Thus, the perception of respiratory distress has a high impact on weaning decisions, whereas the absence of respiratory distress is often overruled by other factors. Given the fact that the large majority of SAS were in the range 0–2, it must be questioned if the SAS is generally rated too low. Judging nasal flaring while infants are on binasal prongs or nose mask is difficult. Thus, actual dyspnea might be underestimated by the assigned SAS. Furthermore, in the clinical routine, the SAS’s interrater reliability was found to be low. This is in contrast with a recent report on the use of the SAS in the delivery room in preterm infants showing good interrater agreement (ICC 0.88), however in this study, the SAS was assigned solely by selected patient care technicians that were trained for the study [22]. In our study, the NICU staff was instructed in the SAS use before its routine implementation, but not directly before or throughout the study. Regular team training using video recordings might help to improve the interrater agreement of the SAS. Currently, there is an observational study investigating the reliability of the SAS in preterm infants (ClinicalTrial Identifier: NCT03199898), and we are eagerly awaiting the results.

Furthermore, weaning decisions are also influenced by so-called soft factors, such as parental attitudes and organization of the NICU team. These factors are often difficult to identify and measure. We analyzed weaning with respect to the weekday and found that infants were more likely to be weaned on Mondays compared to days of the weekend. We assume that this finding relates to the fact that on our ward, weekends are usually covered by an attending neonatologist who may not have been responsible during the week and therefore is more reluctant to change ventilator settings. Furthermore, extreme premature neonates were less likely to be weaned compared to infants born more maturely, despite similar SAS and corrected gestational age at the beginning of the evaluation period. Possibly, healthcare professionals ascribe more importance to the occurrence of other influencing factors and are generally more reluctant to wean in this most vulnerable patient group. These observations call for future studies evaluating the effect of concise weaning protocols that reduce the impact of individual (subjective) judgement by applying objective criteria and restricting “protocol violations” in order to unify the weaning process even in the setting of changing health care professionals especially on the weekends.

Looking at different NIV modes, the only trend towards reducing the length of ventilatory support after implementation of the SAS in routine care was seen for HHHFNC with low PEEP towards the end of NIV. As the infants are usually older when going on HHHFNC, it is conceivable that the before mentioned factors such as apneas and bradycardias occur less frequently, and therefore a low SAS receives more attention in the weaning decision. HHHFNC is tolerated very well by most infants, is easy to handle, and causes less nasal trauma than other devices [23]; therefore, there is little urge to wean. However, despite being considered as more “gentle,” high flow therapy may have adverse effects, including prolonged time on respiratory support and oxygen, more time to full oral feeds, and longer hospitalization [24,25,26]. Therefore, awareness of the factors that (maybe unnecessarily) prolong ventilatory support, and protocols to govern weaning from HHHFNC, are required.

Our study has several limitations. NIV mode and the weaning method were not strictly defined by the study but chosen by the physician in charge, and the SAS was assigned through direct observation of the patient by changing nurses. This pragmatic nature of assessment may be considered a limitation. However, we aimed at evaluating the weaning decisions and the role of the SAS as it is performed in clinical practice instead of creating an “artificial” study setting. Although the rates of different NIV modes were similar, the weaning practice may have changed over time, e.g., more pressure weaning or more time cycling. If one was superior to the other in terms of weaning progress, this might have skewed the results. However, studies comparing different methods so far have not been able to identify the one best weaning approach [27,28].

As a single center, we were only able to include a small number of patients, thus the study was underpowered in detecting small differences in NIV duration. However, the clinical relevance of a slight reduction in NIV days is arguable. Furthermore, we used a historical control group. The groups did not differ for important baseline determinants of respiratory outcomes such as antenatal steroids and postnatal surfactant and caffeine therapy. There were some differences in the incidence of complications that might influence weaning decisions, such as a higher rate of IVH in the control group, whereas PDA occurred twice as often in the prospective cohort. Rastogi and colleagues investigated factors that affect weaning in neonates ≤ 32 weeks of gestation. Neither PDA nor high-grade IVH was independently associated with age at successful weaning in a multivariate analysis stratified by intubation status [29].

## 5. Conclusions

In conclusion, NIV weaning practice is influenced by many factors, and therefore difficult to standardize. A better perception of these factors is important to understand what drives clinical decision making and optimize patient care. Bradycardias were found to play a major role in delaying weaning, however, in view of the possible negative consequences of prolonged NIV, the impact of isolated self-limited bradycardias on patient outcome needs to be considered critically and deserves further investigation. Clinical scores that evaluate the quantity and quality of bradycardias and/or apneas might help to make weaning decisions more objective. The SAS as a measure of respiratory effort is considered important when assessing the need to initiate or continue NIV but is of low predictive value concerning the decision to wean NIV parameters. When using the SAS in routine care, the team should be regularly trained on its use to improve interrater reliability.

## Figures and Tables

**Table 1:** Baseline characteristics and different NIV modes in the study groups.

	SAS Group (n = 33)	Control Group (n = 33)	*p*-Value
Gestational age (weeks)	29.0 (2.2)	29.1 (2.2)	0.90
Birth weight (g)	1267 (314)	1282 (280)	0.84
Female gender (n)	11 (33%)	11 (33%)	>0.99
Cesarean section (n)	28 (85%)	24 (73%)	0.37
Antenatal corticosteroids (n)			0.80
Complete cycle #	21 (64%)	23 (70%)	
Incomplete cycle	6 (18%)	8 (24%)	
None	6 (18%)	2 (6%)	
APGAR			
5 min	8.2 (1.9)	8.3 (2.1)	0.90
10 min	9.3 (0.9)	9.2 (1.3)	0.74
Surfactant * (n)	26 (79%)	22 (67%)	0.41
Intubation * (n)	5 (15%)	3 (9%)	0.71
Caffeine treatment * (n)	30 (91%)	29 (88%)	>0.99
Mode of ventilatory support (n)			
sNIPPV	30 (91%)	27 (82%)	0.47
CPAP	25 (76%)	24 (73%)	>0.99
HHHFNC	23 (70%)	26 (79%)	0.57
HHHFNC with PEEP < 3 cmH_2_O	15 (45%)	20 (61%)	0.32
NAVA	6 (18%)	6 (18%)	n.a.

# two doses of betamethasone 12 mg i.m. 24 h apart; * within 48 h of life; Numerical data are presented as mean (SD) and a *t*-test was used for statistical analysis; Categorical data are presented as n-number (percentage), and a Fisher’s exact test or a Chi-Square test was used, as appropriate.

**Table 2:** NIV outcome parameters.

Number of Days	SAS Group	Control Group	*p*-Value
Total NIV	25 (3–55)	27 (2–69)	0.73
NIV ≥ 30 + 0 weeks CGA	17 (3–45)	20 (2–60)	0.72
(s)NIPPV	5.5 (1–39)	5 (1–47)	0.63
CPAP	4 (1–16)	4 (1–15)	0.80
HHHFNC	12 (3–26)	15.5 (2–35)	0.31
PEEP > 3 cmH_2_O	10 (3–21)	10.5 (2–22)	0.98
PEEP ≤ 3 cmH_2_O	2 (1–13)	5 (1–17)	0.10
Supplemental oxygen	2 (1–62)	3 (1–91)	0.64
Length of hospital stay	48 (24–101)	47 (18–106)	0.74

CGA = corrected gestational age; Data are presented as median (range); A Mann-Whitney test was used for statistical analysis.

**Table 3:** Complications.

	SAS Group	Control Group	*p*-Value
Pneumothorax	5 (15.2%)	3 (9.1%)	0.71
BPD	5 (15.2%)	5 (15.2%)	>0.99
PHT	2 (6.1%)	0	0.49
IVH ≥ 3°	1 (3%)	5 (15.2%)	0.19
Hydrocephalus	0	5 (15.2%)	0.053
PVL	1 (3%)	0	>0.99
PDA	12 (36.4%)	6 (18.2%)	0.17
Sepsis	11 (33.3%)	12 (36.4%)	>0.99
NEC	1 (3%)	0	>0.99
ROP *	3 (9.1%)	7 (21.2%)	0.30

* low grade only due to the exclusion of children with high-grade ROP from trial; BPD: bronchopulmonary dysplasia, PHT: pulmonary hypertension, IVH: intraventricular hemorrhage, PVL: periventricular leukomalacia, NEC: necrotizing enterocolitis, ROP: retinopathy of prematurity; A Fisher’s exact test was used for statistical analysis.

## Data Availability

Data can be obtained upon request from the corresponding author.

## Supplementary Materials

The following supporting information can be downloaded at: https://www.mdpi.com/article/10.3390/children9091292/s1, Supplemental Methods and Figure S1: Bland-Altman Diagram of SAS evaluated by three independent raters.
