# Supplementary material for: Noninvasive Ventilation in Preterm Infants: Factors Influencing Weaning Decisions and the Role of the Silverman-Andersen Score
Source: Children (Basel). 2022 Aug 26;9(9):1292. doi: 10.3390/children9091292 (PMC9497739; doi:10.3390/children9091292)
Supplement: Supplementary file 1 [file children-09-01292-s001.zip › children-1877799-supplementary.pdf]

Evaluation of influence factors and the role of the Silverman-Andersen-Score during weaning of preterm neonates from noninvasive ventilation

Supplemental Methods

Respirators and Interfaces

The following respirators are routinely used on our NICU for noninvasive ventilation of preterm neonates:

- Stephanie (Fritz Stephan GmbH, Gackenbach, Germany)

- Sophie (Fritz Stephan GmbH)

- Servo-i (Getinge Deutschland GmbH, Rastatt, Germany)

In combination with the following interfaces:

- binasal prong (Easy Flow nCPAP Prong; Fritz Stephan GmbH)

- nasal mask (Easy Flow nCPAP Maske; Fritz Stephan GmbH)

For HHHFNC a heated humidifier (MR850; Fisher&Paykel HealthCare GmbH, Schorndorf, Germany) in combination with Optiflow Junior 2 Nasal Cannula (Fisher&Paykel) is used.

Standard initial settings for different NIV modes

	sNIPPV	nCPAP	HHHFNC	NAVA
Respirator/Device	Stephanie/Sophie	Stephanie/Sophie	Fisher-Paykel	Servo-i
PEEP	5 (-7) cmH_2O	5 (-7) cmH_2O	Regulated by flow depending on weight (~5-7 l/min)	5 (-7) cmH_20
PIP	15-20 cmH_20	--	--	Regulated by NAVA-level (1-2 $cmH_2O/\mu V$)
Inspiration time	0.3 s	--	--	--
Frequency	50-60/min	--	--	--
V'max	10-12 l/min (range 6-20 l/min)	10-12 l/min (range 6-20 l/min)	5-7l/min	regulated by respirator (max. 25l/min)
Trigger	External trigger (abdominal respiratory sensor)	n.a.	n.a.	Electrical activity of the diaphragm (Edi)
Trigger level	0.5 (AU)	n.a.	n.a.	0.5 µV

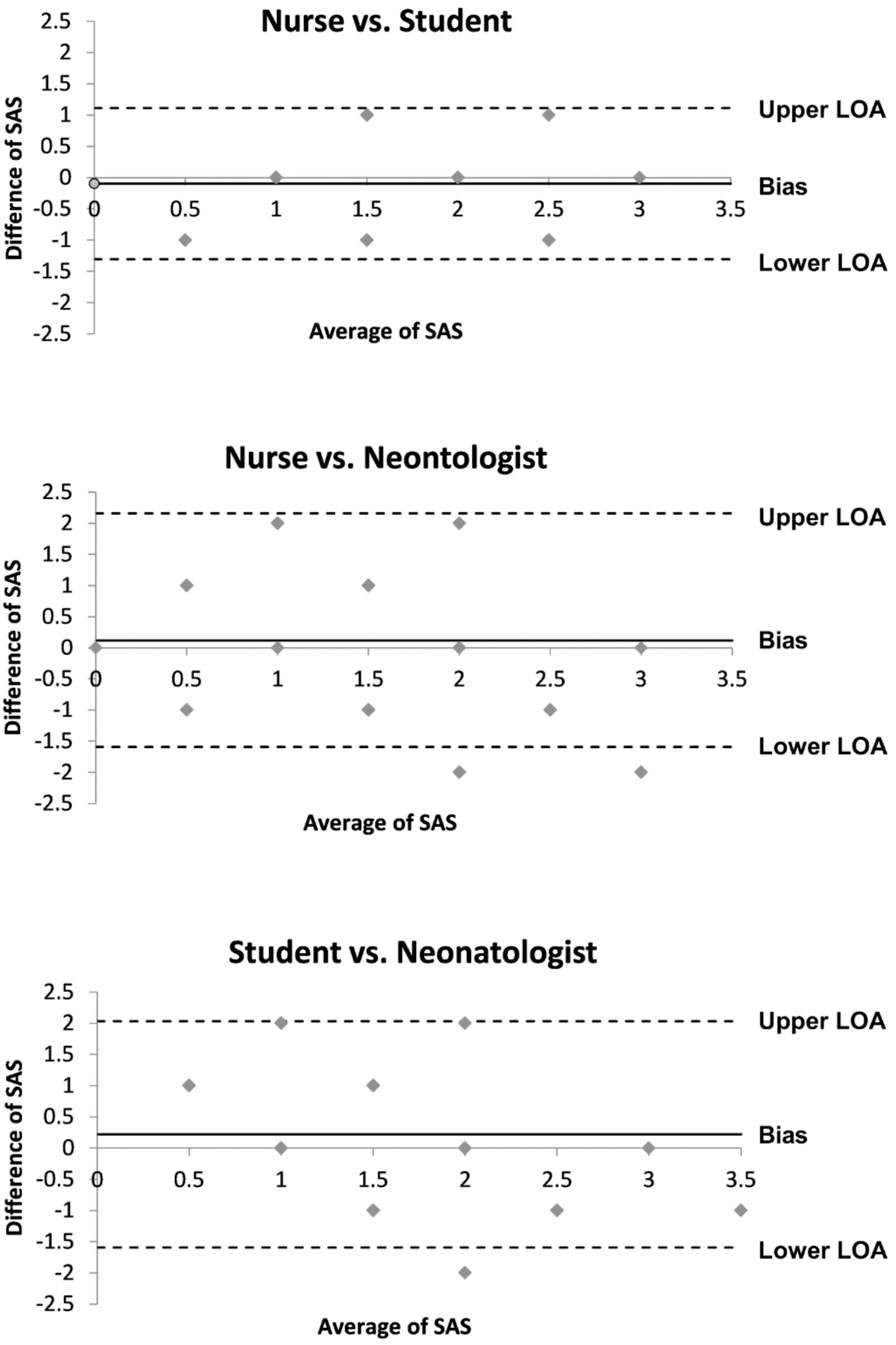

Figure S1) Bland-Altman Diagram of SAS evaluated by three independent raters.
